# Biological Membranes in Extreme Conditions: Simulations of Anionic Archaeal Tetraether Lipid Membranes

**DOI:** 10.1371/journal.pone.0155287

**Published:** 2016-05-11

**Authors:** Luis Felipe Pineda De Castro, Mark Dopson, Ran Friedman

**Affiliations:** 1 Computational Chemistry and Biochemistry research Group (CCBG), Department of Chemistry and Biomedical Sciences, Linnæus University, 391 82 Kalmar, Sweden; 2 Centre of Excellence “Biomaterials Chemistry”, Linnæus University, 391 82 Kalmar, Sweden; 3 Systems Biology of Microorganisms Research Group (SBMR), Centre for Ecology and Evolution in Microbial model Systems (EEMiS), Linnæus University, 391 82 Kalmar, Sweden; University of Leeds, UNITED KINGDOM

## Abstract

In contrast to the majority of organisms that have cells bound by di-ester phospholipids, archaeal membranes consist of di- and tetraether phospholipids. Originating from organisms that withstand harsh conditions (e.g., low pH and a wide range of temperatures) such membranes have physical properties that make them attractive materials for biological research and biotechnological applications. We developed force-field parameters based on the widely used Generalized Amber Force Field (GAFF) to enable the study of anionic tetraether membranes of the model archaean *Sulfolobus acidocaldarius* by computer simulations. The simulations reveal that the physical properties of these unique membranes depend on the number of cyclopentane rings included in each lipid unit, and on the size of cations that are used to ensure charge neutrality. This suggests that the biophysical properties of *Sulfolobus acidocaldarius* cells depend not only on the compositions of their membranes but also on the media in which they grow.

## Introduction

Extremophiles are microorganisms (including archaea) that optimally live under conditions that are extreme to most other organisms, such as low- or high-pH, non-ambient temperatures, and very high salt concentrations. There is vast interest in understanding how extremophiles can survive in such harsh environments [1]. One of the reasons for this interest in extremophiles is the similarity between the conditions in which extremophiles such as *Sulfolobus acidocaldarius* live (temperatures nearing 80°C, and low pH) and the conditions under which early life may have evolved on Earth some 3.45 billion years ago [2]. Moreover, such conditions are more likely to be relevant for life on other planets or their satellites.

The membranes of extremophiles are the first barrier between the organism and the harsh environment and are therefore well-suited for living under such harsh conditions [3]. Their extreme durability is desired for biotechnological applications, e.g., as a means for drug delivery [4]. The ability to sustain a wide range of temperatures and pH is to a large extent due to the physicochemical properties of the archaeal membranes. Understanding of the physical properties of archaeal membranes and how they are coupled to their chemical compositions is therefore of interest from both a purely scientific and a technological point of view.

Data on the atomistic structure of lipid membranes is hard to come by. The fluidity of the membranes makes it difficult to study them by X-ray diffraction and their hydrophobicity limits the applicability of structural methods such as NMR. With the advancement of computer power, algorithms, and methods, the use of computer simulations has become a convenient alternative [5–9]. Such simulations require a long process of equilibration, which can be circumvented by use of existing models (i.e., pre-equilibrated structures, taken from long simulations that were run in the past). Such models are available for some model phospholipid membranes such as 1,2-dioleoyl-*sn*-glycero-3-phosphocholine (DOPC) and 1,2-dipalmitoyl-*sn*-glycero-3-phosphocholine (DPPC). Archaeal cells are surrounded by di-ether and tetraether membranes, rather than phosphodiesthers, and simulations of such membranes had been few and far between.

In an early attempt to model tetraether membranes, Gabriel and Chong have modelled membranes composed of glycerol dialkylnonitol tetraether (GDNT) lipids from the thermoacidophilic archaea *Sulfolobus acidocaldarius*. The simulation suggested that GDNT membranes are much more tightly packed than ester-linked bilayers [10]. Although the conclusion most probably holds true, these early simulations were rather short and were carried out under T = 450K and *in vacuo*. Nicolas studied asymmetric tetraether membranes in solution and compared their properties to DPPC membranes simulated under the same conditions [11]. His conclusions (based on sub-ns simulations that are considered very short today) were that the archaeal monolayers are characterised by slow dynamics. Shinoda and co-workers used 1,2-di-O-phytanyl-*sn*-glycero-3-phosphocholine (diphytanyl phosphatidylcholine, DPhPC), 1,2′-O-biphytanyl-1′,2-di-O-phytanyl-*sn*-diglycero-3,3′-bisphosphocholine (acyclic tetraether phosphatidylcholine, a-TEPC), and tetra-O-di-(biphytanyl)-*sn*-diglycero-3,3′-bisphosphocholine (macrocyclic tetraether phosphatidylcholine, m-TEPC) to model di- and tetra-ether membranes and studied their dynamics using 25 ns long simulations. They analysed the effects of cyclic and non-cyclic lipids on the membrane structure and used free energy calculations to analyse water permeation [12]. The same authors later studied how diether bilayer membranes interact with ions in NaCl solutions [13]. They concluded that the archaeal lipid membranes are highly stable against salt as they show minor changes in their physical properties with increasing salt concentrations.

Interest in simulations of archaeal membranes has increased in recent years, owing to biophysical characterisation of various such lipids and biotechnological interest. Marrink and co-workers used coarse-grained computer simulations for the design of robust membranes that mimic archaeal structures [14]. Efremov and co-workers also studied model tetraether lipids, using atomistic zwitterionic models that were carried out over a wide range of temperatures [15]. Finally, Tarek, Miklavčič and co-workers have used small angle X-ray scattering and computer simulations to study membranes of the halophilic and extremely thermophilic archaea *Aeropyrum pernix* [16]. Their study revealed that the archaeal lipid bilayers were less hydrated than conventional phosphatidylcholine lipids, and not affected by salt.

**Fig 1:**
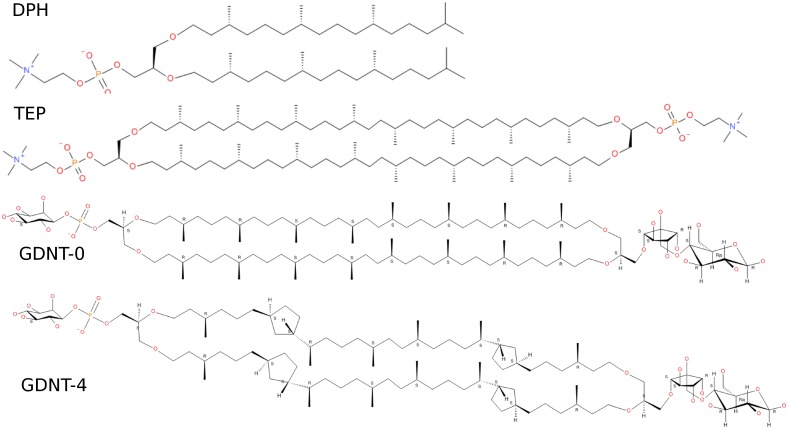
Chemical structures of the di- and tetra-ether lipids simulated in this study. DPhPC (DPH), ether-diphytanylphosphatidylcholine. TEP, di-O-biphytanyphosphatidylcholine. GDNT, glycerol dialkylnonitol tetraether, with zero (GDNT-0) or four (GDNT-4) cyclopentane rings.

*S. acidocaldarius* is a biotechnologically important microorganism owing to its ability to withstand low pH, high temperatures, and the presence of toxic metals [17, 18]. Various biochemical and biophysical mechanisms ensure the organism’s survival in such harsh conditions and its unique membrane structure is certainly significant in this respect. The major component of the plasma membrane of *S. acidocaldarius* and other thermoacidophilic archaea is bipolar tetraether lipids (about 90% of the total lipids in *S. acidocaldarius*) [10]. The bipolar tetraether lipids mixture is comprised of 10:1 glycerol dialkylnonitol tetraether (GDNT, Fig 1) and glycerol dialkylglycerol tetraether (GDGT). GDGT and GDNT consist of a pair of 40-carbon phytanyl hydrocarbon chains, in which branched methyl groups and 0–4 cyclopentane rings are present (the higher the temperature, the higher the number of rings) [19]. Atomistic studies of GDNT membranes have been previously performed [10] but only over very short time scales. Thus, we have developed modern force-field parameters for GDNT membranes that are compatible with the widely-used general Amber force field (GAFF) [20, 21] and the GROMACS program [22–24] (of note, other modern membrane force-field, namely CHARMM [25] and GROMOS [26] enable accurate simulations of membranes). We use those parameters to simulate GDNT membranes with and without embedded pentameric rings and compare their biophysical properties to these of diether and plain tetraether membranes (for which paramaters have also been developed). Finally, we examine the effects of cations of two different sizes on the membrane packing.

## Methods

### Building and preparation of membrane structures for MD simulations

**Modeling and parameterization of di- (DPhPC) and tetraether (TEP) reference phospholipids** In contrast to standard diester lipids for which pre-equilibrated bilayer models exist and are used in most published studies, no pre-equilibrated models of such membranes were available for simulation of di- and tetra-ether membranes. A first model for the GDNT-[0, 4] monomer was built on the basis of suitable templates. 1,2-diphytanoyl-*sn*-glycero-3-phosphocholine (ester diphytanoylphosphatidyl-choline, ester-DPhPC) was selected as a template and was modified into the respective diether lipid (ether-DPhPC, DPhPC). The stereochemistry of chiral centres (asymmetric glycerol, C2 atom and branched carbons), all-*R*, was set according to the structural formula depicted in Fig 1. This structure was then converted into 3D by means of the CORINA software [27] and the torsion angles were adjusted in order to obtain a conformation suitable for building a bilayer model with di-O-alkyl chains roughly parallel to each other and the PC head group bent in approximately the same plane by transferring them from the conformation of one (out of 72) DOPC single lipid from a 100-ns snapshot available for download [21, 28].

The 3D structure of the bipolar, macrocyclic tetraether lipid, di-O-biphytanyphosphatidyl-choline (TEP) was built by linking the tails of the 3D structure of two DPhPC molecules with a conformation more suitable to build a membrane model (see above). The resulting stereochemistry of the chiral centres of the biphytanyl chains was adjusted according to that in the snapshots of MD simulations of TEP membrane models kindly provided by Wataru Shinoda, which correspond to the models described in ref. [12].

In the next step, DPhPC and TEP were set up using GAFF parameters according to the procedure described in ref. [21] for DOPC. After fixing atom numbering and naming of the models, the program antechamber [29] of the AmberTools software suite (version 12) was used to derive GAFF parameters, i.e., for the preparation of input files for the Amber program LEaP using the bcc charge model. After checking that all of the needed force field parameters were available, the corresponding Amber topology and coordinates as well as unit library files were generated.

Amber files were converted to the GROMACS compatible Amber force-field [30] using the script amb2gmx.pl. Monovalent ions and a TIP3P water box were added to a single DPhPC molecule in the Amber tool xleap, and Amber topology and coordinate files were created for this unit and converted into GROMACS files. Trial simulations were carried out with a single DPhPC molecule in an aqueous solution, which ensured the usability of the input files.

**Modeling and parameterization of GDNT-0 and GDNT-4 bipolar tetraether lipids** Molecular models of GDNT-0 and GDNT-4 with the proper head groups were built and prepared for simulations. The ether linkages in the glycerol backbones of these natural archaeal lipids are characterised by an exceptional *sn*-2,3 configuration contrary to *sn*-1,2-diacylated glycerol in bacteria and eukaryotes (used also in DPhPC and TEP lipids mentioned above). The unusual configuration (*R* without head group) at the *sn*-2 position of these lipids was achieved through inversion of chirality of the corresponding, asymmetric glycerol C atom of TEP.

To model the actual composition of the head groups of the archeal lipids, the PC moieties in TEP were replaced in a further step by myo-inositolphosphate on one side of the molecule and by the five-ring of calditol attached to *D*-glucopyranose on the other. Two cyclopentane rings were incorporated at each biphytanyl chain of the macrocycle with their proper stereochemistry set. GDNT membranes are modelled with all charges on the same side of the membrane, corresponding to experimental data [31, 32].

After both Amber systems were set up with GAFF similarly to DPhPC and TEP, one counter ion was added near to the negatively charged phosphoinositol head group of each GDNT molecule for electroneutrality required for MD simulations and Amber library and coordinate (pdb) files were generated.

**Building of DPhPC, TEP, GDNT-0 and GDNT-4 membrane models in water** Bilayer and monolayer models in pure water were built out of 72 single DPhPC or 36 bipolar tetraether lipids (TEP, GDNT-0 and GDNT-4, the latter two with 36 counter ions as neutralising charges), respectively, and 2088 water molecules (SPC/E model, as recommended for use with GAFF parameters) were arranged in a rectangular box using the software PACKMOL [33]. The Lateral box dimensions were set to reach an area per lipid (APL) of approximately 70 (TEP) or 100 (GDNT) Å^2^ to facilitate relaxation, with 50 (60) Å in the z direction, for DPhPC and TEP (GDNT), and 10 Å for each water slab. These membrane models were set up with GAFF parameters using the lipid library files created previously. They were then converted into GROMACS files.

### Molecular dynamics simulations

A first series of MD simulations of the hydrated tetraether TEP membrane model (monolayer) and the reference, diether lipid DPhPC membrane model (bilayer) were set up and carried out with the aim of designing a suitable protocol for follow-up MD simulations of the more elaborated membrane models. All simulations were carried out using GROMACS [22–24], version 4.5.5.

First, an unrestrained energy minimization (EM) was performed starting with the DPhPC and TEP membrane models, by use of the steepest descent minimizer to remove close contacts and two different EM protocols. The energy profiles displayed smooth convergence up to a maximum force less than 1000 kJ mol ^-1^nm ^-1^. No distortion of the bi- or monolayer structure or the water slabs was observed. The final structures were very similar to the starting ones. Altogether, the system was sufficiently stable to proceed with the MD simulations.

The setup chosen for the equilibration and data collection phases of the MD simulation at constant temperature: T = 325 K (TEP, DPhPC and GDNT-0) or T = 350 K (GDNT-0 and GDNT-4). For GDNT-0 with one type of counter ions (A^+^) an additional simulation at 325 K was carried out to be compared with that of TEP at the same temperature. The simulations were carried out under constant pressure in the direction of the membrane normal (1 bar) and constant surface tension (*γ* = 44 mN/m, where *γ* is the total surface tension of the system); i.e., mimicking the NP*γ*T ensemble, as recommended for GAFF [21]. The equilibration of the membrane began by use of position restraints on the heavy atoms of lipid molecules during the initial phase, while allowing the water molecules to rearrange freely. The restraints were gradually released within 600 ps simulation time. In the case of GDNT membrane models the temperature was gradually increased in 50 K steps each 50 ps up to the target T = 350 K corresponding to the optimal growth temperature mentioned above. A weak temperature and pressure coupling (Berendsen) [34] was used with a time constant of 0.1 ps for temperature coupling (with the lipids and aqueous solvent, together with ions if present, explicitly coupled separately) and 0.5 ps for pressure coupling with isothermal compressibility of 4.5⋅10^-5^ bar^-1^. Subsequently, in a second step of approximately 16 ns, further equilibration cycles using a more accurate temperature coupling method (modified Berendsen thermostat, V-rescale [35]) and an increased time constant (0.5-3.0 ps for pressure coupling) were performed. Further MD settings included periodic boundary conditions, PBC, in all directions, and holonomic constraints (LINCS [36]) with all bonds constrained with lincs_iter = 1 and lincs_order = 4. The integration time step was 2 fs. The neighbour list was updated every 5 steps. A 1.2 nm short-range cutoff was used for the neighbour list, electrostatics, and van der Waals interactions. Long range electrostatic forces were approximated by particle mesh Ewald [37] with cubic interpolation (pme_order = 4) and a grid spacing for FFT (Fourier spacing) of 0.16 nm. Dispersion correction was also applied. Fluctuations and drift of the values of the APL, volume per lipid (VPL) and repeat distance (Lz) gradually decreased within 35 ns (Figures A and B in S1 File) and data analysis was carried out from the remaining 70–80 ns of the simulations. Extending the simulations of TEP (Figure C in S1 File) and GDNT-0 by 100 ns reveals that the systems do not exhibit large modification in any of those properties after 35 ns.

After the time required for equilibration was established, multiple simulations were carried out and proper statistical measurements made: three additional, independent 70-80 ns trajectories were recorded for each system starting from the last snapshot of the equilibration phase.

GDNT membranes may interact with different ions under biological or chemical set-ups. Here, we did not wish to study any particular cation, but rather simulate the effect of having relatively small and hard cations (that would correspond to alkali cations such as K^+^) versus larger and softer cations (that would correspond to multi-atomic ions such as ammonium, but without considering the additional complexity of specific atomic interactions). To this end, two sets of counterions were used, small (A^+^), and large (B^+^), to examine the effect of size of the counter ions on the membrane properties (see Table 1 for the force field parameters). For GDNT-0 with A^+^ counter ions an additional simulation at 325 K was carried out to be compared with that of TEP at the same temperature and to be able to assess the combined effect of lipid head groups and the presence of neutralising charges on the results. Parallel simulations of GDNT-4 in pure water using two different target values of the surface tension (*γ* = 24 and 64 mN/m) were performed and the effect on the calculated APL investigated (see S2 File).

**Table 1:** *σ* and *ϵ* Lennard Jones parameters for ions.

Ion	*σ* (nm)	*ϵ* (kJ mol^-1^)
A^+^	0.418758	0.543502
B^+^	0.543217	0.418400

### Calculation of the biophysical properties of membranes

The biophysical properties of membranes that are calculated here include the area per lipid (APL), volume per lipid (VPL), volume per membrane unit (VMU), bulk modulus expansion (*κ*_*A*_), and two measures of the membrane thickness, namely peak-to-peak distance of the electron density profile (d_PP_) and repeat distance(Lz).

The APL is calculated as the lateral area of the simulation box (the *xy*-plane) divided by the number of lipids in one lipid leaflet or monolayer surface. VPL and Lz were calculated from the box dimensions (*box*_*x*_, *box*_*y*_, *box*_*z*_) according to the formulas [38]:

$$\begin{array}{c} V_{box}=N_{L}\times VPL+N_{W}\times V_{W} \end{array}$$ (1)

$$\begin{array}{c} L_{z}=\frac{V_{box}-N_{W}\times V_{W}}{box_{x}\times box_{y}} \end{array}$$ (2)

Where *V*_*box*_ is the volume of the simulation box; *N*_*L*_ and *N*_*W*_ are the numbers of lipid and water molecules in the simulation box, respectively; and *V*_*W*_ is the volume of a water molecule simulated under the same conditions separately (1728 water molecules, NPT ensemble with isotropic pressure coupling). *V*_*W*_ is 30.41Å^3^ at 325 K and 30.97Å^3^ at 350 K.

The bulk modulus expansion was determined from the APL probability distribution as calculated from the time evolution of APL during the sampling phase according to the equation [39]:

$$\begin{array}{c} \kappa_{A}=\frac{kT\langle A\rangle }{N\langle \delta A^{2}\rangle } \end{array}$$ (3)

where *k* is the Boltzmann constant, *T* is the absolute temperature, 〈*A*〉 is the average APL, *N* is number of lipid molecules in a bilayer leaflet or on one side of the membrane spanning monolayer, and 〈*δA*^2^〉 corresponds to mean squared fluctuations of the APL.

The membrane thickness can be assessed from the peak-to-peak distance (d_PP_) for the peaks of the electron density profiles corresponding to the position of the head groups. This property was then used to calculated the VMU, by multiplying the average values of d_PP_ and the APL:

$$\begin{array}{c} VMU=\langle A\rangle \;\cdot \;\langle d_{PP}\rangle \end{array}$$ (4)

### Visualisation of membrane structures

Figures of the membranes were created with DS-visualizer (Accelrys, Inc).

## Results and Discussion

### Equilibration of the initial membrane structures

Accurate simulations of lipid membranes require long phases of equilibration. Using a controlled equilibration protocol (see the Experimental section), actual analysis was started after approximately 35 ns. It was apparent that the APL and repeat distance (Lz) display long-term fluctuations with a frequency of several nanoseconds and similar amplitude, whereas the VPL was more stable (Figures A and B in S1 File). For this reason, it was necessary to analyse the data over tens of nanoseconds. We note that earlier attempts (by others) to model TEP, DPhPC, and GDNT membranes relied on much shorter simulations, probably due to computational limitations.

To examine whether the simulations are indeed equilibrated, the drift in the APL, VPL and Lz of the TEP membrane were followed for a further 100 ns (Figure C in S1 File). The averages and fluctuations of these properties were indeed very similar to those obtained over the shorter time scales, which indicated that sampling over 70-80 ns was sufficient.

Fluctuations of the APL and VPL were larger for GDNT (Figure D in S1 File). However, analysis of multiple simulations revealed that the systems could be analysed after 35 ns of simulation time, as before. For systems containing GDNT lipids, fluctuations in the values of the APL could be as long as 20 ns (Figure E in S1 File). The values of the APL and VPL levelled off when equilibrated over a long time (Figure F in S1 File). The fluctuations remained even after an extension of 100 ns simulation time, indicating that they were a feature of the system’s dynamics. Analysis was therefore performed for simulations of 70-80 ns, beginning approximately at t = 35 ns.

### The structure of DPhPC and TEP membranes

**Fig 2:**
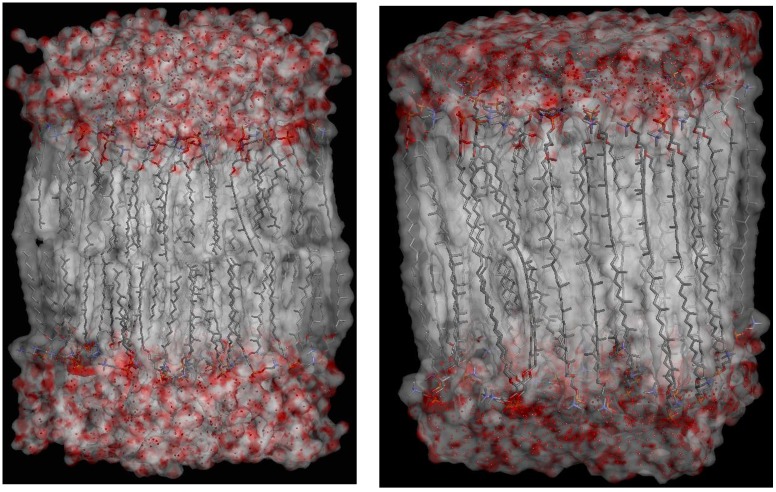
Simulation structures. Structures of a DPhPC bilayer (left) and a TEP monolayer (right). Only a single unit is displayed.

**Fig 3:**
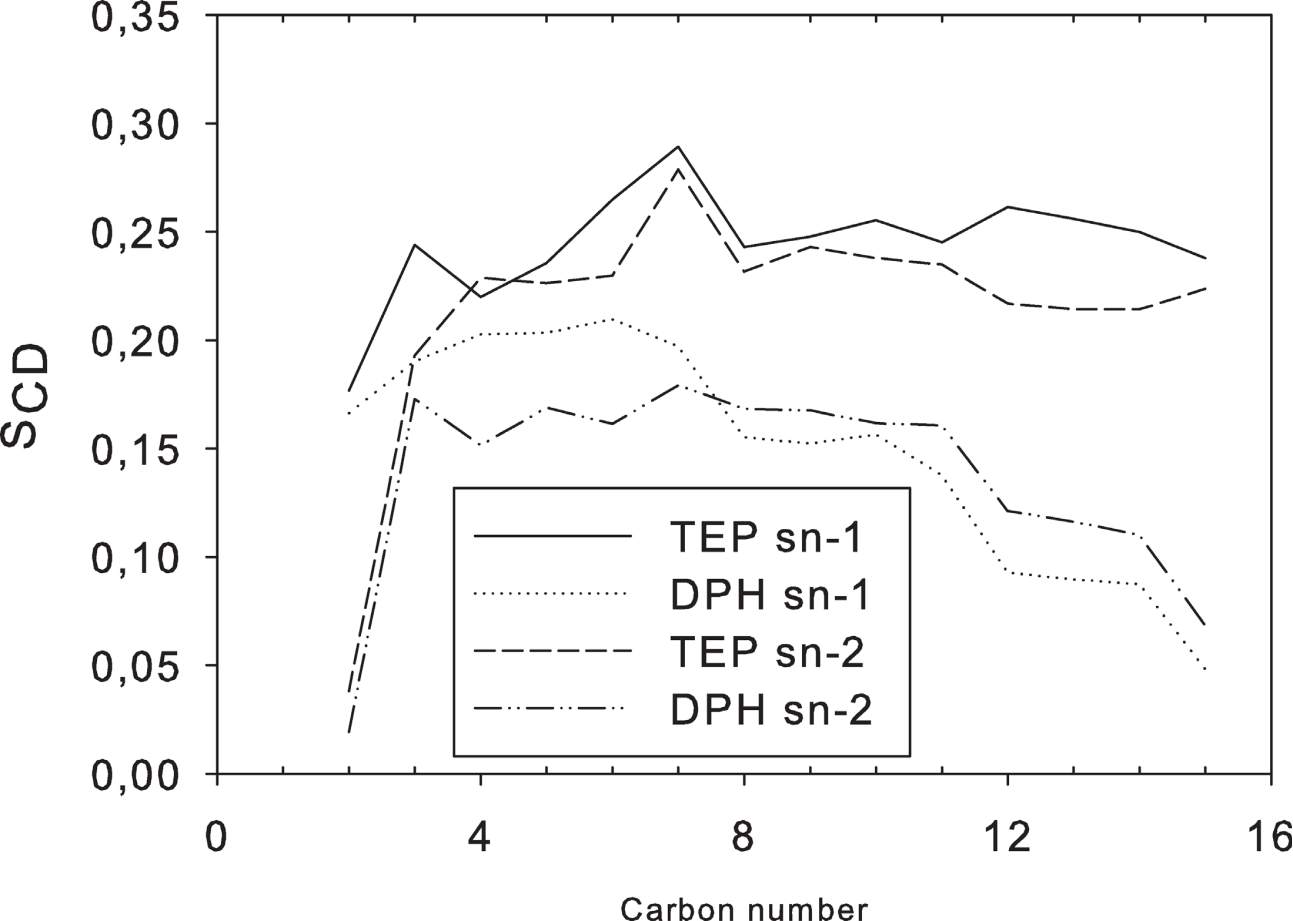
Deuterium order parameter (S_CD_) calculated along the main lipid hydrocarbon chains (*sn*-1/2) of DPhPC and TEP membrane models.

Under the simulation conditions (T = 325 K, p = 1 bar), DPhPC membranes maintained a liquid-crystalline, bilayer structure (Fig 2). TEP membranes were liquid-crystalline monolayers that appeared somewhat more ordered (Fig 2). This was also evident from the calculated deuterium order parameters (Fig 3A). In comparison with the DPhPC membranes, TEP membranes are characterised by larger APL and shorter peak-to-peak distance of the electron density profile (d_PP_) which represented the distance between the phosphate groups on each side of the membrane (Table 2). In both cases, simulations with our GAFF parameters yielded APL, VPL, and d_PP_ values that were somewhat smaller than those obtained by simulations with the CHARMM force field [12]. Differences between these results and the earlier simulations with the CHARMM27 force field [40] may also be due to sampling, as the membranes were simulated and the values averaged over a longer period in this study. The VPL was also larger for the DPhPC membranes. Smaller variations between DPhPC and TEP membranes were observed with respect to the repeat distance (Lz) and membrane-unit volume. Of note, Shinoda and co-workers have used their own software, MPDyn [41], for their simulations.

**Table 2:** Physical properties of the simulated membranes.

	T (K)	APL (Å^2^)	VPL (Å^3^)	Lz(Å)	d_PP_(Å)	VMU (Å^3^)	*κ*_*A*_ (dyn cm^-1^)
DPhPC	325	72.4(.4)	1390(14)	38.4(.6)	38.0(.4)	2746(1)	1126(720)
DPhPC, data from [12]	353	74.3	NA	NA	38.2	2834	670
TEP	325	67.4(.1)	2667(9)	39.6(.2)	40.2(.4)	2708(.6)	5187(1553)
TEP, data from [12]	353	70.2	NA	NA	39.2	2752	2020
GDNT-0 (A^+^)	325	71.7(.1)	2937(9)	41.0(.2)	39.9(.9)	2863(1)	5141(1970)
GDNT-0 (A^+^)	350	71.1(.2)	2971(17)	41.8(.4)	37.7(1.1)	2678(1)	2604(681)
GDNT-0 (B^+^)	350	76.4(.3)	3047(19)	39.9(.4)	36.8(1.1)	2810(2)	2210(903)
GDNT-4 (A^+^)	350	67.2(.2)	2863(15)	42.6(.3)	40.2(.8)	2701(1)	4948(3556)
GDNT-4 (B^+^)	350	70.9(.1)	2974(11)	41.6(.2)	37.9(.4)	2683(.7)	4262(785)

Values are average over four independent trajectories, with error estimates (in parentheses) based on block averages over five blocks, or standard deviation (for bulk modulus expansion, *κ*_*A*_). Data for tetraether membrane with zero (GDNT-0) and four (GDNT-4) cyclopentane rings were calculated with two model counter-ions, smaller (A^+^) and larger (B^+^), see the text for details. All data were calculated from multiple trajectories (n = 4). NA = not available.

### Rigidity and flexibility of tetraether phospholipid membranes

**Fig 4:**
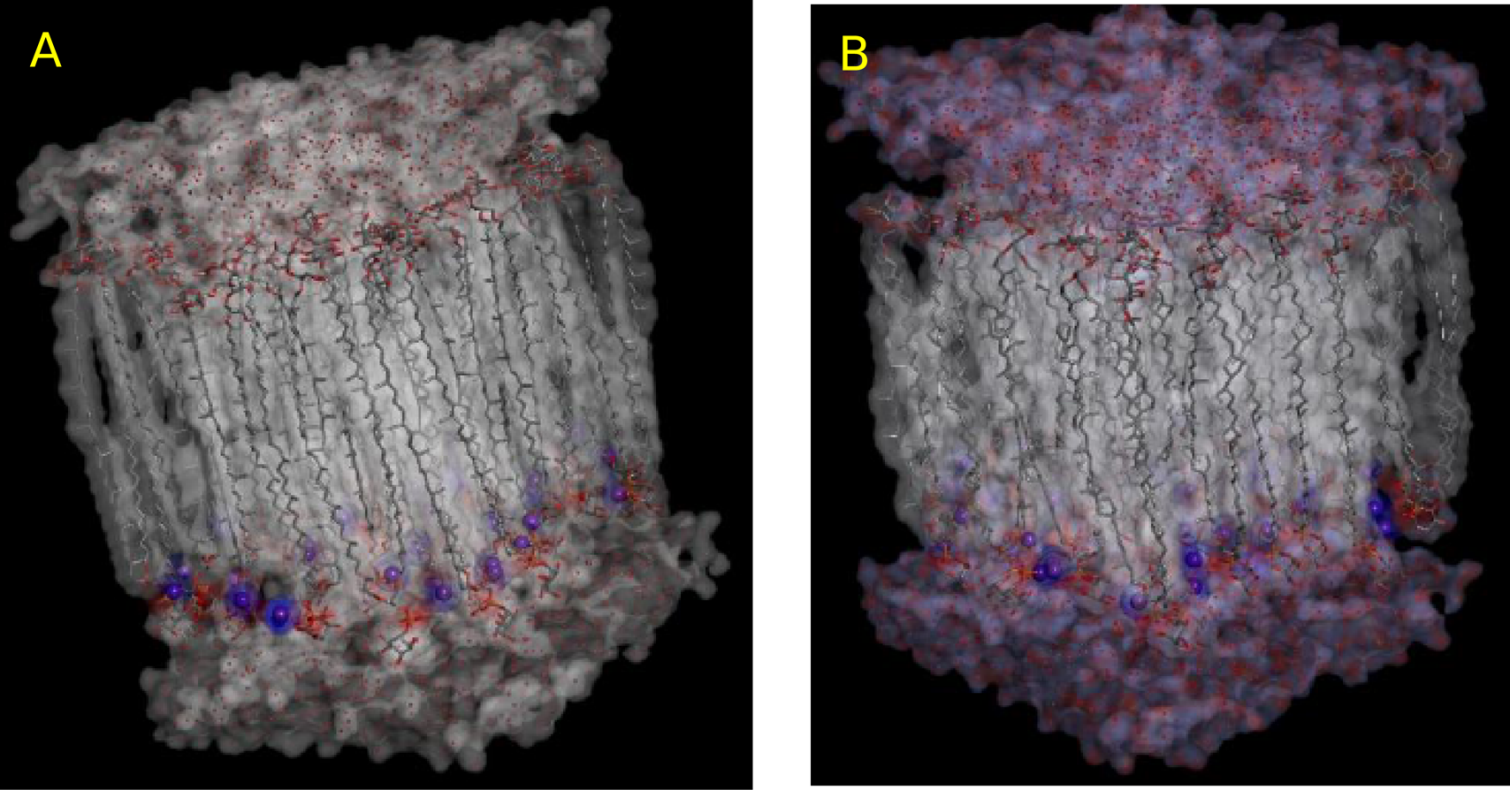
Simulation structures. Structures of (A) GDNT-0 and (B) GDNT-4. Only a single unit is displayed.

Extremophiles are able to function over a wide range of temperature, from below zero [42] to 122°C [43]. In the absence of cholesterol or non-saturated lipids in their membranes, they adjust their membrane flexibility by incorporating cyclopentane rings into the tetraether lipid units. We therefore simulated tetraether phospholipid membranes without (GDNT-0) or with four (GDNT-4) cyclopentane rings embedded in each lipid unit (Fig 1). Using the developed force-field parameters, GDNT-4 formed more flexible liquid-crystalline structures at T = 350K (Fig 4). Some physical properties of the membrane are summarised in Table 2.

In spite of the ordered structure of the GDNT-0 membranes, some differences were observed as a function of temperature between T = 325K and T = 350K. At the higher temperature, GDNT-0 membranes had a slightly *lower* volume per membrane unit (VMU) despite similar VPL and APL, which indicated that they were able to fit together more tightly when the temperature was increased. The VMU was calculated by multiplying the peak-to-peak distance (d_PP_) for the peaks of the electron density profiles corresponding to the position of the phosphate / glucose head groups. Indeed, d_PP_ is larger for GDNT-0 membranes simulated at 325 K. In comparison to GDNT-0 membranes, GDNT-4 membranes had smaller area and volume per lipid, and they were broader, having larger repeating units (Lz) and d_PP_.

**Fig 5:**
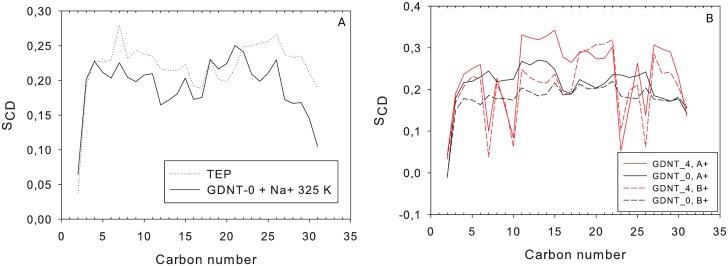
Deuterium order parameter (S_CD_) calculated along the main lipid hydrocarbon chains (*sn*-1/3) of GDNT membrane models. (A) A comparison between GDNT-0 at T = 325K and TEP. (B) A comparison between GDNT-0 and GDNT-4, with different counter-ions.

The deuterium order parameter along the main hydrocarbon lipid chain showed a similar profile, but overall higher values in TEP than GDNT-0 (chain *sn*-1/3, Fig 5A). The bulkier head groups of GDNT seemed to lower the overall segment order of the chains. Interestingly, GDNT-0 and GDNT-4 membranes displayed a different order parameter profile (Fig 5B), mainly due to carbon atoms of the cyclopentane rings, especially those on the ring corners—C7, C10, C23 and C26. These atoms displayed lower S_CD_ values. Other GDNT-4 carbon atoms displayed higher order parameters in comparison with the ring-free system.

The calculated value of the elastic area expansion modulus for the TEP monolayer was approximately three times higher than that for the DPhPC bilayer, as reported in Table 2. This finding was in par with the pioneering study of Shinoda *et al*., who attributed the higher tensile strength of the TEP membranes to their cyclic structure. The effect of lipid hydrocarbon chain tail-to-tail cyclization on the flexibility of membrane area expansion, i.e., on its tensile strength and stability to external forces could be confirmed by the present study. However, the absolute values of *κ*_*A*_ obtained by us were much higher than in ref. [12]. This could be related to the larger dispersions calculated for a shorter (25 ns) simulation by Shinoda and co-workers and/or the different simulation conditions (NPT with anisotropic pressure coupling versus NP*γ*T ensemble here). Moreover, *κ*_*A*_ strongly depends on the surface tension [39]: the lower is the surface tension, the higher is *κ*_*A*_. GDNT-0 membranes had *κ*_*A*_ values that were smaller than those of TEP but larger than those obtained with DPhPC. Including rings in GDNT lipids increased their tensile strength, as evident when GDNT-0 and GDNT-4 membranes were compared.

### The fine structures of the di- and tetraether membranes

**Fig 6:**
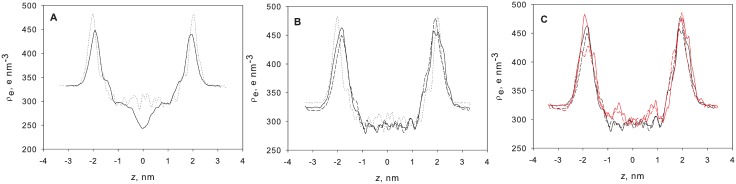
Total electron density profiles of the simulated membrane models along the membrane normal. The centre of the membrane corresponds to z = 0. (A) DPhPC bilayer (solid line) vs. TEP monolayer (dotted line); (B) GDNT-0 monolayers with small (A^+^, solid line) and large (B^+^, dashed line) counter ions vs. TEP monolayer (dotted line); (C) GDNT-4 monolayers with small (solid red line) and large counter ions (dashed red line) vs. GDNT-0 monolayers with small (solid line) and large (dashed line) counter ions.

The electron density profiles of the membrane models were calculated to further characterise the membranes’ atomistic structures (Fig 6). The electron density profiles of the DPhPC bilayer and the TEP monolayer models were very similar to those reported in the literature [12, 16]. The bilayer showed the usual shape with a trough in the centre corresponding to the slip plane between the two lipid layers. TEP and GDNT membranes were monolayers and showed no such trough, but rather small fluctuations of electron density in this region. TEP membranes displayed higher and sharper peaks at the PC head group position than the DPhPC bilayer. The sharpness of the peaks indicated a flatter surface [12].

In comparison to TEP, the total electron density profile of the GDNT-0 membranes, as depicted in Fig 6B, displayed lower and less sharp peaks at the head group positions. The phosphate head groups were shifted slightly to the inside in comparison with TEP membranes, forming a somewhat thinner membrane (d_PP_ = 37.7 / 36.8 vs 40.2 Å). The thickness of the GDNT-0 membrane, as measured by repeat distance calculated from the box volume, Lz, was 41.8 / 39.9 Å depending on the size of the counter ions. These values were very similar to those obtained for TEP (39.6 Å). The probable reason for the discrepancy between the two measures of membrane thickness (d_PP_ and Lz) was the different definition of membrane thickness. The different head groups of the lipids seemed to affect their exposure to the water and thereby both Lz and d_PP_.

**Fig 7:**
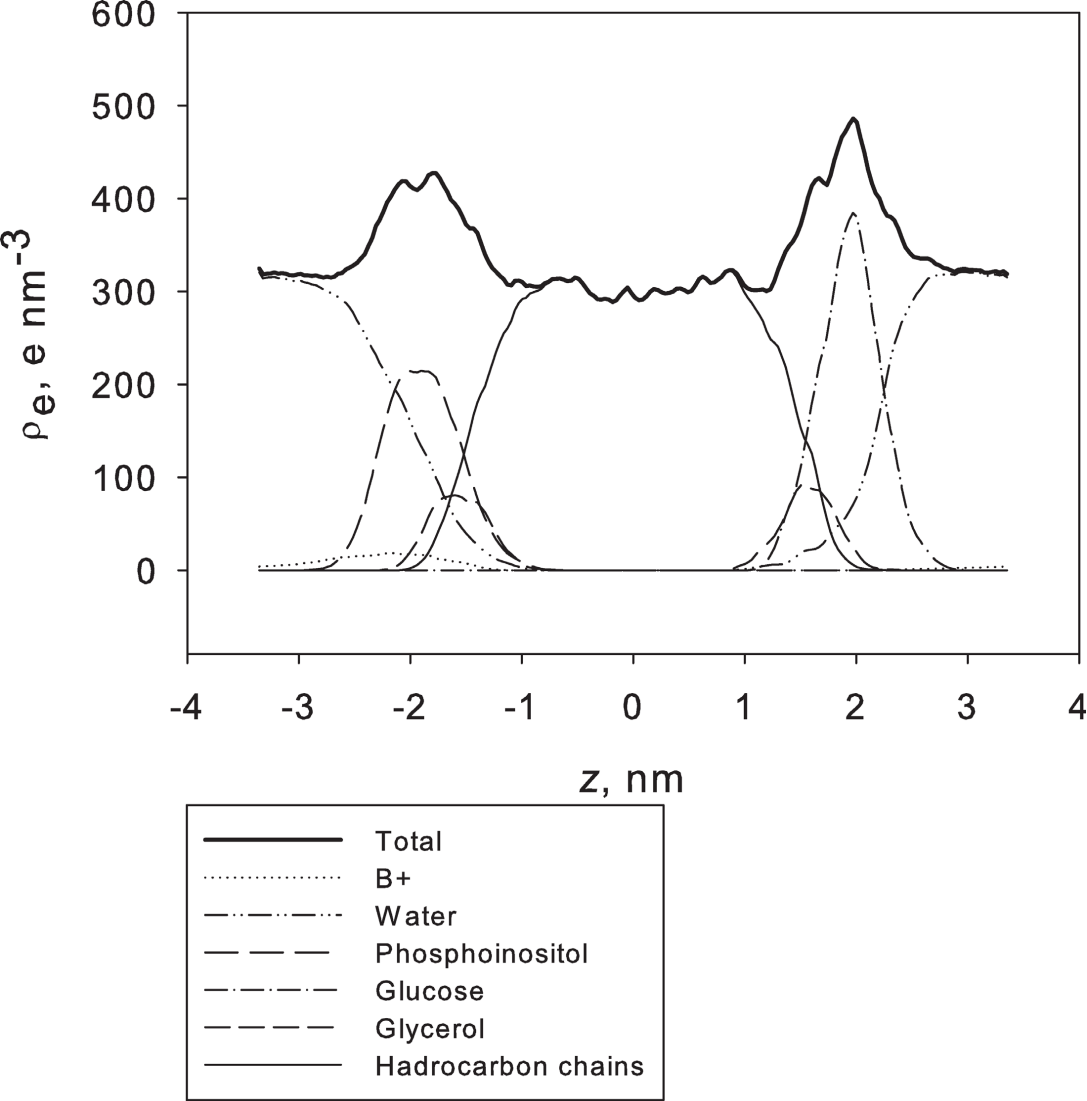
Total and separate electron density profiles of GDNT-4 with neutralising charges. The inset depicts the density of the B^+^ ions.

As shown in Fig 6C, the total electron density profiles for both the GDNT-0 and GDNT-4 membrane models were quite similar. GDNT-4 displayed a higher electron density and larger fluctuations in the membrane centre region (near z = 0). The peaks at the head group positions, especially for phosphoinositol, were higher for GDNT-4 chains than for the linear lipids, but both membranes displayed similar peak widths. The membranes formed by GDNT-4 lipids were slightly thicker than or as thick as the GDNT-0 monolayers both according to d_PP_ and Lz. The contributions of different constituent groups (phosphoinositol and glucose head groups, glycerol backbone, hydrocarbon chains as well as water and counter ions) were plotted in Fig 7 for GDNT-4. A shallow peak corresponding to counter ions overlapping with the phosphoinositol and glycerol electron density peaks could be observed. This peak diminished almost completely in the region of the glucose head groups.

### Interactions of the membranes with neutralising charges

GDNT lipids are anionic, and were simulated in the presence of counter ions added to neutralise the overall charge. To examine the effect of these counter ions, cations of two different sizes, small (LJ radius of approximately 0.21 nm) and large (LJ radius of approximately 0.27 nm) were used, and their interactions with the membranes were examined. Such specific interactions can influence how lipid vesicles, and even archaea cells, behave in different media. The larger ions were considered to be somewhat softer, i.e., they had smaller absolute values for the LJ potential energy depth, *ϵ* (Table 1). We used the symbols A^+^ for the small ions and B^+^ for the larger ones, that can represent soluble organic cations that are still fairly simple in structure and contain just a few atoms.

**Fig 8:**
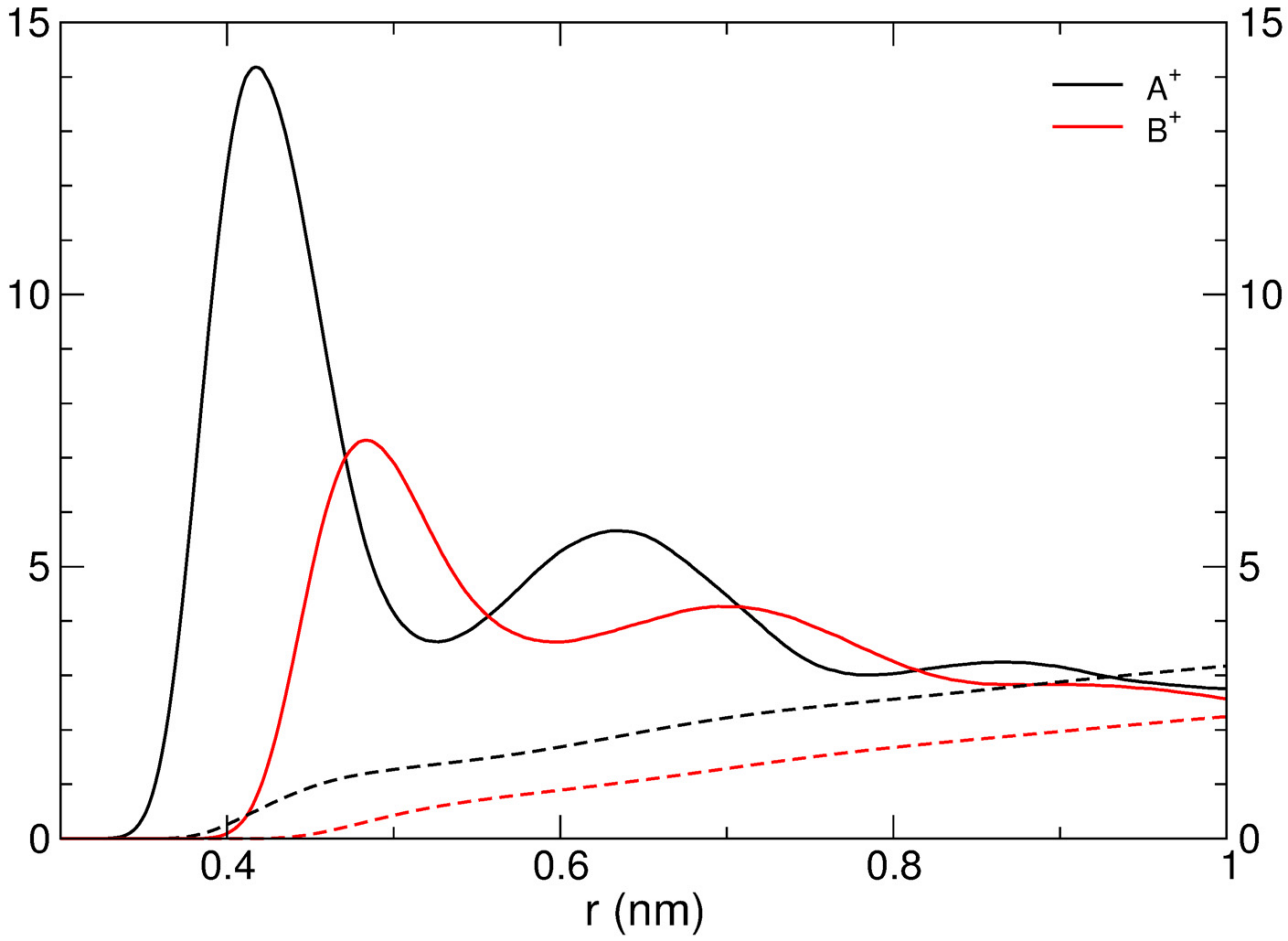
The radial distribution of counterions around the lipid phosphate ions, calculated from simulations of GDNT-4 membranes. The cumulative number of ions at a given distance from the phosphate ions is shown by the dashed lines. A value of 1 for the radial distribution function indicates the bulk distribution. For the cumulative number, values in the Y-axis are the actual number of ions within a given distance from a phosphate group.

The APL and VPL of the membranes were larger when the simulations were performed in the presence of the larger counter ions (Table 2 and Figure D in S1 File). This may be explained by a higher affinity between the smaller ions and the phosphate groups (as shown previously for negative residues in proteins and counter ions [44]). Examination of the ion-phosphate radial distribution function lead to the same conclusion. The smaller ions had a much larger probability to form a closed sphere complex with the phosphate group, i.e., to lose their solvation shell completely (Fig 8). This led to stronger electrorestriction [45], and thereby smaller structures. Thus, when preparing vesicles from GDNT, the choice of salt is likely to affect the physical properties of the membranes (previous studied have already considered the effects of temperature and pH [46–48]). The effect of the counter ions can be comparable to that of rings in the membranes in some aspects (compare the GDNT-0 system with smaller ions and the GDNT-4 system with larger ions, Figure D in S1 File).

**Fig 9:**
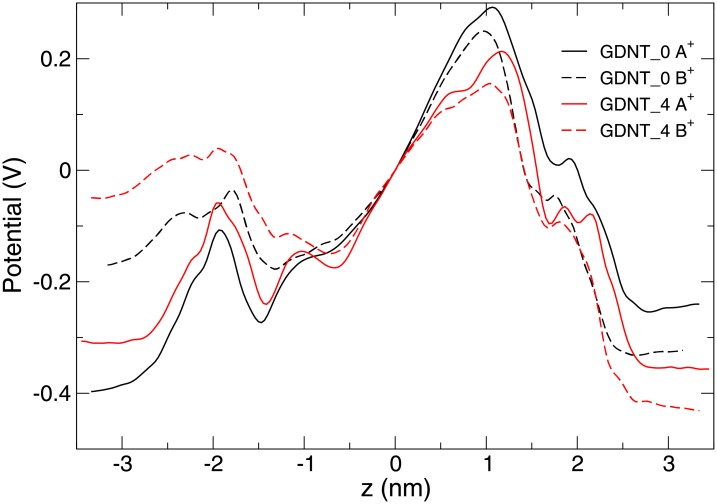
The electric potential across the membrane. The potential is shown over the z axis for the four different simulations. The charged phosphate groups are located on the left-hand side of the figure (lower values of z). The potential is calculated with reference to the centre of the membrane, where the charge is roughly zero. Each line is an average over four simulations, where the calculation was carried out over 40 ns. The membranes are centred at x = 0 nm. Note that the (average) box length is not exactly the same due to the simulation conditions (constant pressure, variable volume).

The electrostatic potential across the membranes is shown in Fig 9. The profile is different than the more uniform potential observed in simulations of model anionic membranes composed of phosphatydilserine [49]. The asymmetry of the monolayers results in a potential that is not symmetric as well. The potential exhibits two peaks. The peak on the left-hand side is due to binding of the ions and is more pronounced for the simulations where the smaller neutralising charges (A^+^) were used. All peaks are slightly less pronounced in the GDNT-4 membranes that include cyclopentane rings. This is apparently due to the reduced order at the rings (Fig 5).

The GDNT membranes are asymmetric, which is manifested in the order parameters, electron density profiles and electrostatic potential. These effects may be somewhat different in experimental systems, that are several orders of magnitude larger than the cells simulated here, and where the membrane can accommodate to forces operated on it by bending. Finite size effects in simulations of membranes have been extensively discussed, and are sometimes rather small [50]. On the other hand, anti-symmetrical systems are more prone to size effects, especially when ion-binding is considered [51]. In our system, membrane charges are concentrated on one side, but so are the ions (Fig 7, inset) and we expect that bending will be less significant than biological aspects such as the presence of various (non-GDNT) lipids, proteins and other biomolecule as part of the archaeal membrane. Thus, we have preferred longer sampling and running of multiple simulations to a study involving a large membrane unit (that will not only take longer to simulate but also to equilibrate).

### Comparison of the properties of archaeal and phospholipid membranes

It is interesting to compare the physical properties of the archaeal membranes simulated here with those calculated for phospholipid membranes. Some widely used force fields for phospholipids include GAFF [21], which we used here; CHARMM36 [52]; Gromos54A7 [53]; and Lipids14 (based on the AMBER force field) [54]. These force fields yield physical properties (such as e.g., APL, d_PP_ and order parameters) that are within few percent of the experimental values, and thus form a good basis for comparison.

Considering the most common phospholipids that are studied and simulated, namely DLPC, DMPC, DPPC, DOPC, POPC and POPE, these membranes have APL in the range of 55.5 or 59.2 Å^2^ (POPE, values for Lipid14 and CHARMM36, respectively) to 69.0 Å^2^ (DOPC). Our results reveal that the diether lipid membranes have APL as large as that of DOPC if not larger (67.4–76.4 Å^2^, Table 2). Previous studies have shown that the value of the APL is sensitive to the ensemble (NPT / NP*γ*T) and degree of solvation [52]. Here, it is shown that GDNT membranes, the nature of the counter-ion may also contribute.

The volumes per lipid for PC and PE membranes are also available from the literature, and range from 949 (DLPC) to 1250 Å^3^ (DOPC) with Lipids14. Diether membranes have larger volume per membrane unit (1390 Å^3^). Tetraether membranes take twice as much volume per membrane unit because they are monolayers and each lipid is roughly twice the size of a phospholipid monomer. Peak-to-peak distance (d_PP_) of the archaeal membranes (36.8–40.2 Å) is within the range of values observed for phospholipid membranes except the thinner DLPC and DMPC [54].

Interestingly, we noticed that the APL and VPL of GDNT-membranes were larger when the simulations were performed in the presence of the larger counter ions. A similar observation was reported for a combination of 4:1 POPC/POPS phospholipids, where the APL increased from 54.6 (with Na^+^) to 56.1 (with K^+^) and 60.7 Å^2^ (with Cs^+^) [55]. Of note, the POPC/POPS with Alkali ions were run with a fairly high ion concentration (1M) in addition to neutralising charges. In the same study, it has also been reported that membranes simulated with Cs^+^ ions had lower d_PP_, whereas membranes simulated with Na^+^ or K^+^ ions always had similar thickness. Here, we noticed that the d_PP_ was shorter when GDNT membranes were simulated with larger counter ions. NMR studies of DOPC and DOPG membranes did not reveal structural changes in zwitterionic DOPC membranes in the presence of 0.3M NaCl or 0.1M CaCl_2_ [56]. However, interactions between the ions and anionic DOPG membranes were observed in the same study.

## Conclusions

We developed GAFF parameters for di- and tetraether membranes, including TEP, DPhPC, and GDNT from *S. acidocaldarius*. Analysis of simulations revealed that all membrane structures were stable, with the GDNT-4 membranes more rigid than the corresponding GDNT-0 ones. Our simulations suggested that GDNT-0 membranes were larger than GDNT-4 membranes in terms of their areas and volumes, and had a fairly ordered structure, whereas GDNT-4 membranes were more resistant to tension.

Interestingly, although *S. acidocaldarius* can tolerate different salts, GDNT membranes do undergo some changes upon binding to different types of counter-ions. The larger the ion, the larger the area and volume per lipid. This indicated the influence of specific ion effects [57] that depended on the charge density of the ions, at least under ambient salt concentrations. Interestingly, in terms of the area and volume per lipid, the effect of ions could be almost as large as the effect of including cyclopentane rings in the structure. Archaea can respond to a change of environmental conditions (temperature) by tuning membrane composition. Thus, it is reasonable that such organisms are likely have different membrane compositions when they live (or are grown) in media that contain different cations.

A limitation of computational studies such as this is that, at present, the biophysical properties of archaeal membranes are not well-characterised. Thus, a comparison to experimental data (which can be tricky in any case [58]), is not possible here. A combined experimental-theoretical effort will be required to assess and improve the current force-field.

## Supporting Information

S1 File Supplementary figures. A supplementary file containing Figures A–F.
(PDF)

S2 File Appendix—effect of the surface tension parameter on the properties of the membranes. An appendix file containing text and a table.
(PDF)
