# Supplementary material for: Biological Membranes in Extreme Conditions: Simulations of Anionic Archaeal Tetraether Lipid Membranes
Source: PLoS One. 2016 May 11;11(5):e0155287. doi: 10.1371/journal.pone.0155287 (PMC4864297; doi:10.1371/journal.pone.0155287)
Supplement: S1 File — A supplementary file containing Figures A–F. (PDF) [file pone.0155287.s001.pdf]

Electronic Supplementary Information for:
Biological Membranes in Extreme Conditions: Simulations of Anionic Archaeal Tetraether Lipid Membranes

Luis Felipe Pineda De Castro [1,2,¤], Mark Dopson[3], Ran Friedman [1,2,*]

1 Computational Chemistry and Biochemistry research Group (CCBG), Department of Chemistry and Biomedical Sciences, Linnæus University, 391 82 Kalmar, Sweden
2 Centre of Excellence "Biomaterials Chemistry", Linnæus University, 391 82 Kalmar, Sweden
3 Systems Biology of Microorganisms Research Group (SBMR), Centre for Ecology and Evolution in Microbial model Systems (EEMiS), Linnæus University, 391 82 Kalmar, Sweden

¤Present address, Laboratory of Molecular Modeling, University of Gdansk, ul. Wita Stwosza 63, PL-80-308 Gdansk, Poland

*** ran.friedman@lnu.se**

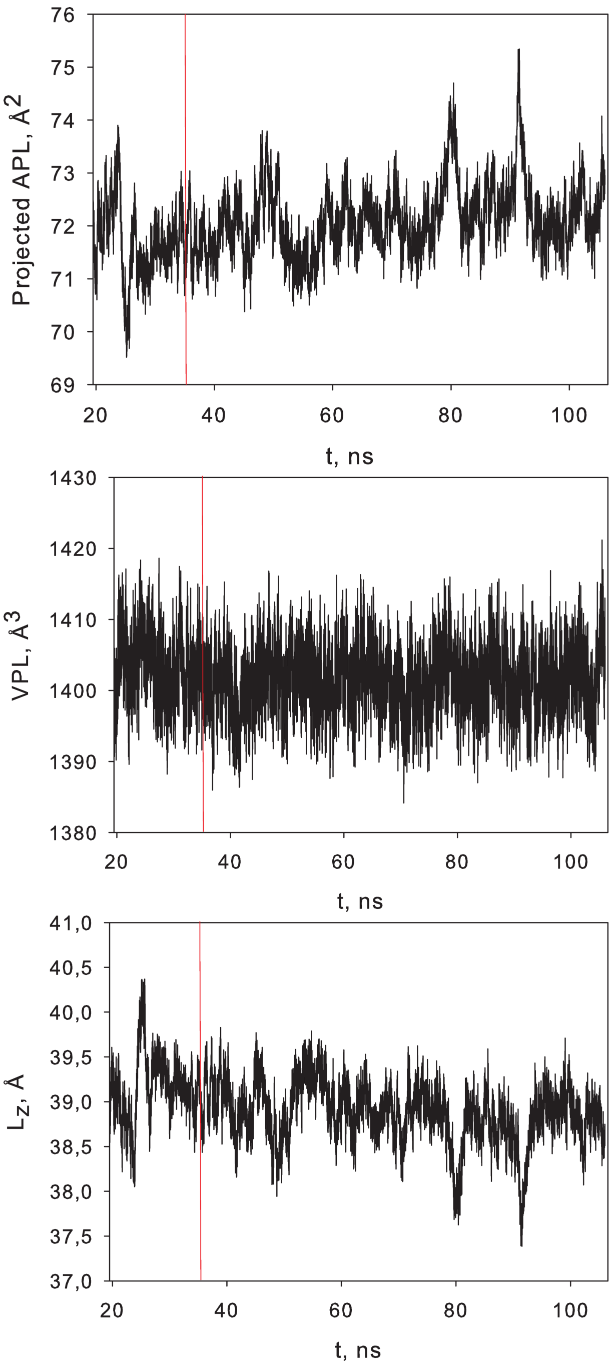

Figure A. Time evolution of projected area per lipid (APL), volume per lipid (VPL), and repeat distance (Lz) for DPhPC bilayer. Data analysis of the simulation starts at the time point marked by a red vertical line.

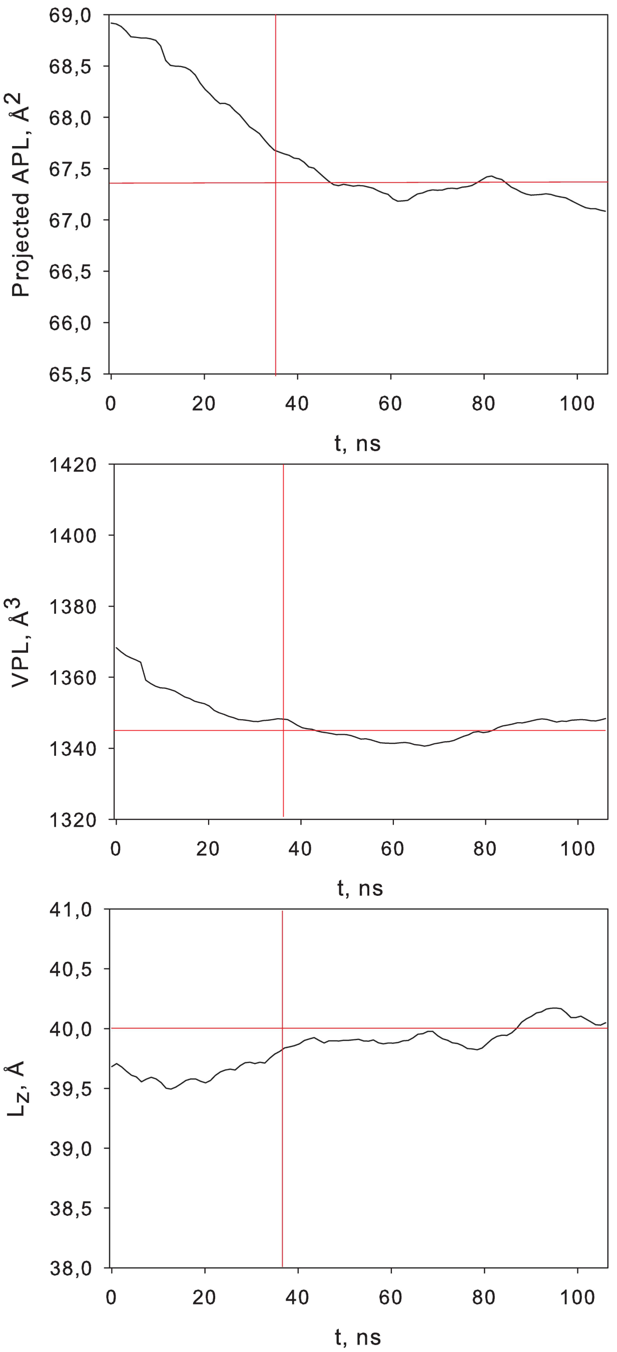

Figure B. Time evolution of projected area per lipid (APL), volume per lipid (VPL), and repeat distance (Lz) for the TEP membrane. The values are running averages. Data analysis of the simulation starts at the time point marked by a red vertical line. The VPL values in the figure are halved to allow a comparison with bilayers. The actual values are given in Table 2.

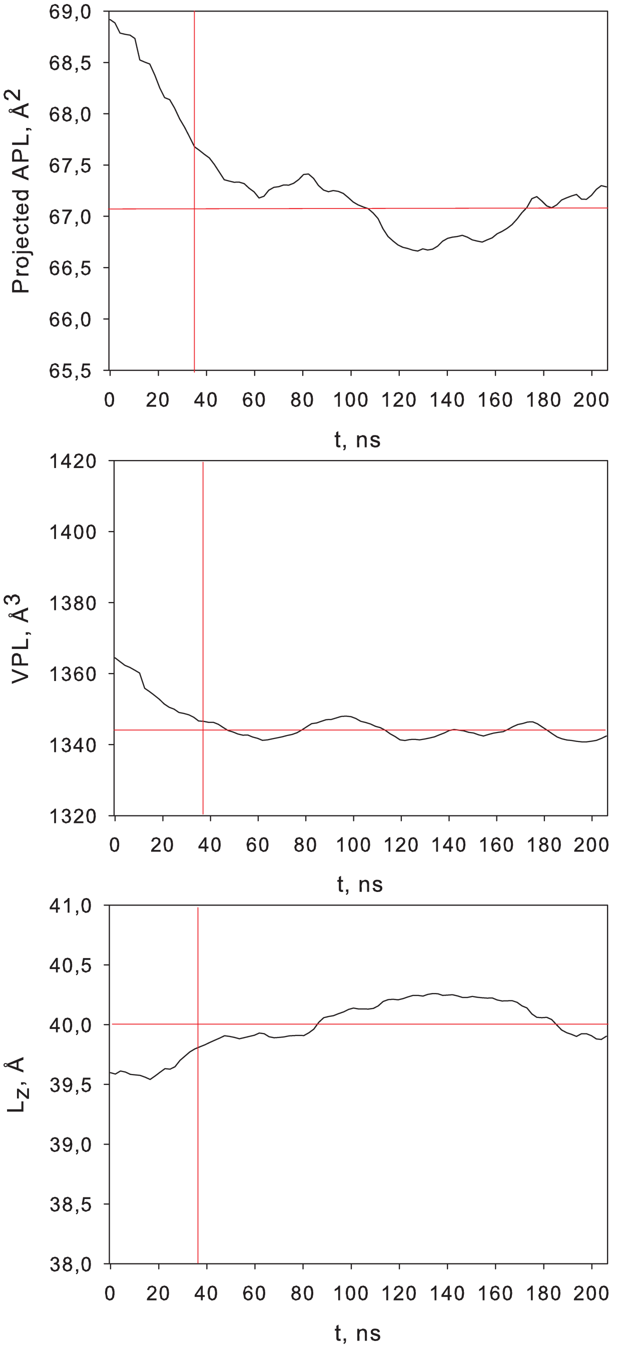

Figure C. Elongation of the simulation of TEP. Time evolution of projected area per lipid (APL), volume per lipid (VPL), and repeat distance (Lz) for the TEP membrane. Data analysis of the simulation starts at the time point marked by a red vertical line. The VPL values in the figure are halved to allow a comparison with bilayers. The actual values are given in Table 2.

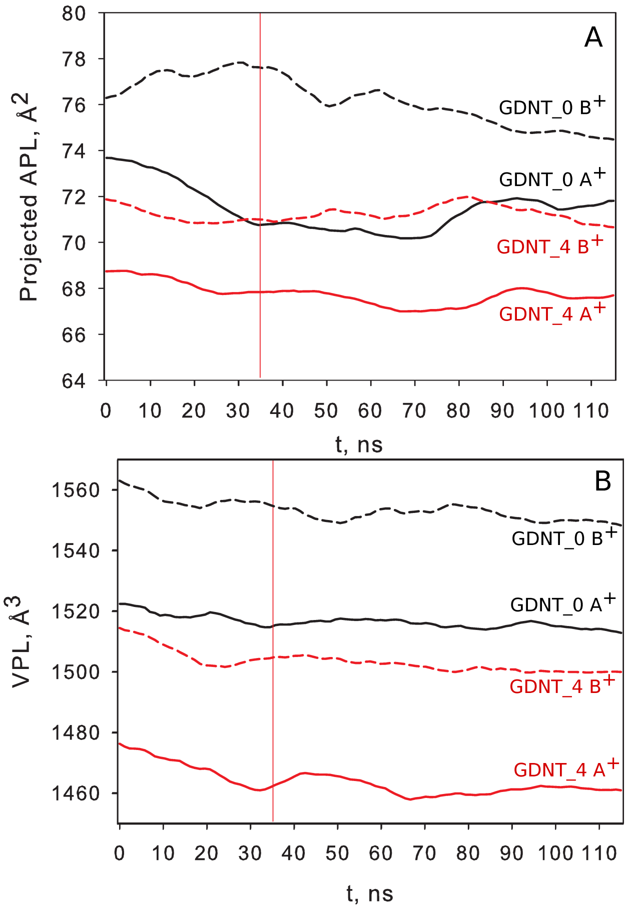

Figure D. Time evolution of (A) projected area per lipid (APL) and (B) volume per lipid (VPL) of the GDNT membranes. The values are running averages. Data analysis of the simulation starts at the time point marked by a red vertical line. The VPL values in the figure are halved to allow a comparison with bilayers. The actual values are given in Table 2.

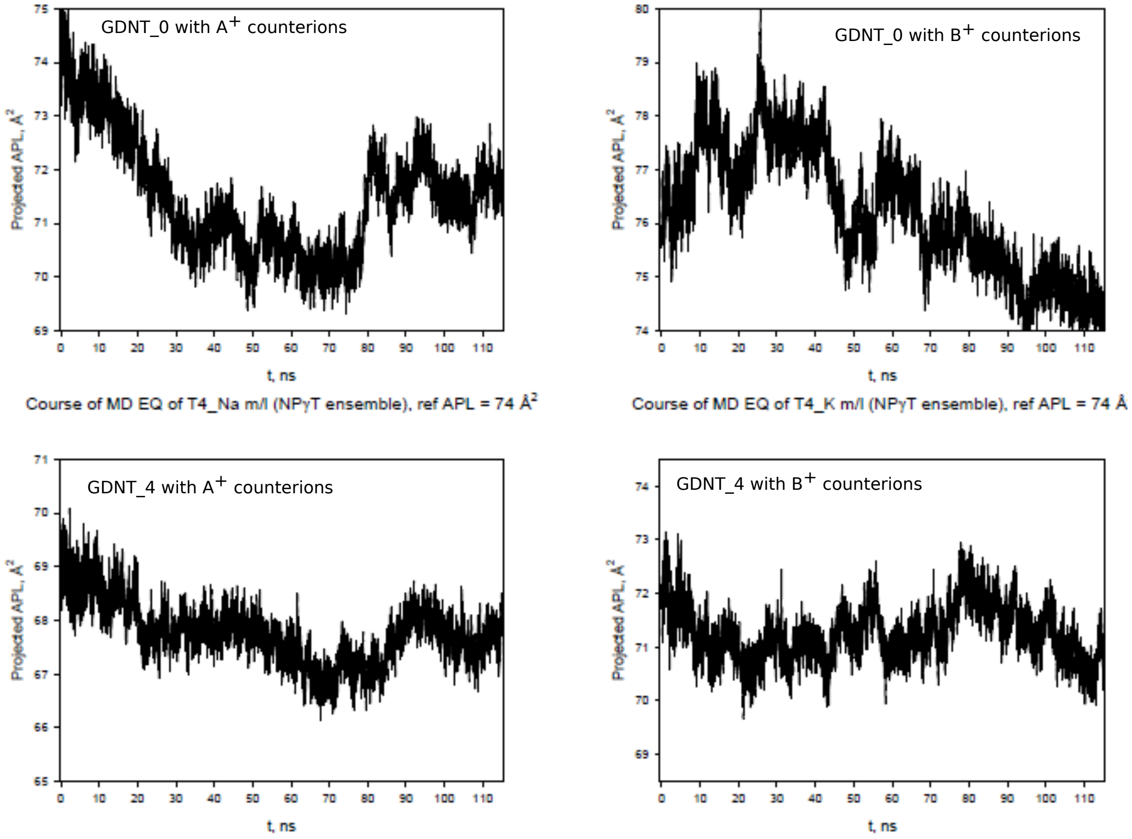

Figure E. Time evolution of the projected area per lipid (APL) of GDNT-0/4 monolayers with two types of counterions: small (A^+) and large (B^+).

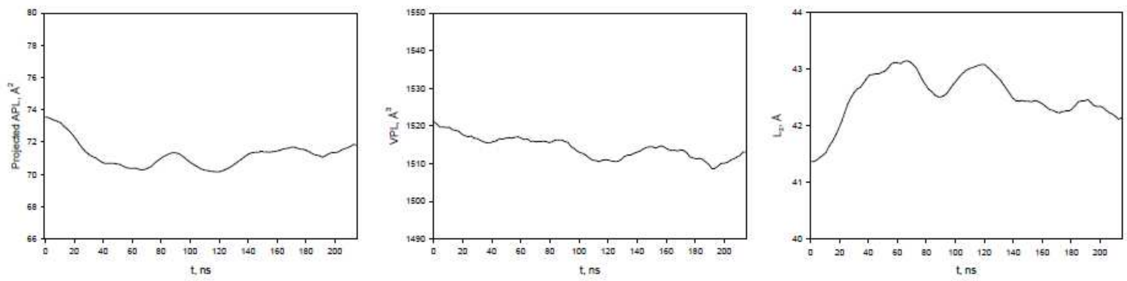

Figure F. Time evolution of the APL, VPL and Lz of GDNT-0 monolayers with small counterions.
