# Supplementary material for: Biological Membranes in Extreme Conditions: Simulations of Anionic Archaeal Tetraether Lipid Membranes
Source: PLoS One. 2016 May 11;11(5):e0155287. doi: 10.1371/journal.pone.0155287 (PMC4864297; doi:10.1371/journal.pone.0155287)
Supplement: S2 File — An appendix file containing text and a table. (PDF) [file pone.0155287.s002.pdf]

Appendix for:

Biological Membranes in Extreme Conditions: Simulations of Anionic Archaeal Tetraether Lipid Membranes

Luis Felipe Pineda De Castro [1,2,¤], Mark Dopson[3], Ran Friedman [1,2,*]

1 Computational Chemistry and Biochemistry research Group (CCBG), Department of Chemistry and Biomedical Sciences, Linnæus University, 391 82 Kalmar, Sweden
2 Centre of Excellence "Biomaterials Chemistry", Linnæus University, 391 82 Kalmar, Sweden
3 Systems Biology of Microorganisms Research Group (SBMR), Centre for Ecology and Evolution in Microbial model Systems (EEMiS), Linnæus University, 391 82 Kalmar, Sweden

¤Present address, Laboratory of Molecular Modeling, University of Gdansk, ul. Wita Stwosza 63, PL-80-308 Gdansk, Poland

*** ran.friedman@lnu.se**

Appendix - effect of the surface tension parameter on the properties of the membranes

All membrane simulations were carried out using a set-up that mimicked the NpγT ensemble, i.e., constant number of particles, pressure, temperature and surface tension. The surface tension of di- and tetra-ether membranes was not known from experiment, and an ambient value of 44 mN/m [1] was used in the simulations. To examine the effect of altering the surface tension, simulations of the GDNT_4 system with B^+ counter ions were also performed at reduced (24 mN/m) and increased (64 mN/m) surface tension. The results showed little dependence of membrane properties on the exact value of the surface tension. Deviations between the systems were smaller than 5% in all of the calculated observables (Table 1).

Table 1. Effect of the surface tension (γ) on the physical properties of GDNT-4 membranes

γ (mN/m)	APL (Å^2)	VPL (Å^3)	Lz(Å)	d_{PP}(Å)	VMU (Å^3)
24	67.8(.1)	2933(10)	43.3(.2)	39.0(.4)	2647(.6)
44	70.9(.1)	2947(10)	41.6(.2)	37.9(.4)	2683(.7)
64	70.6(.2)	2947(12)	41.7(.3)	38.8(.4)	2741(.6)

References

1. Siu SW, Vácha R, Jungwirth P, Böckmann RA. Biomolecular simulations of membranes: physical properties from different force fields. J Chem Phys. 2008;128:125103.
